# Supplementary material for: Large-scale two-photon imaging revealed super-sparse population codes in the V1 superficial layer of awake monkeys
Source: eLife. 2018 Apr 26;7:e33370. doi: 10.7554/eLife.33370 (PMC5953536; doi:10.7554/eLife.33370)
Supplement: Transparent reporting form [file elife-33370-transrepform.docx]

***eLife’s* transparent reporting form**

We encourage authors to provide detailed information *within their submission* to facilitate the interpretation and replication of experiments. Authors can upload supporting documentation to indicate the use of appropriate reporting guidelines for health-related research (see [EQUATOR Network](http://www.equator-network.org/%20)), life science research (see the [BioSharing Information Resource](https://biosharing.org/)), or the [ARRIVE guidelines](http://www.plosbiology.org/article/info:doi/10.1371/journal.pbio.1000412) for reporting work involving animal research. Where applicable, authors should refer to any relevant reporting standards documents in this form.

If you have any questions, please consult our Journal Policies and/or contact us: [editorial@elifesciences.org](mailto:editorial@elifesciences.org).

**Sample-size estimation**

- You should state whether an appropriate sample size was computed when the study was being designed
- You should state the statistical method of sample size computation and any required assumptions
- If no explicit power analysis was used, you should describe how you decided what sample (replicate) size (number) to use

Please outline where this information can be found within the submission (e.g., sections or figure legends), or explain why this information doesn’t apply to your submission:

In non-human primate research, the standard is to use two animals. Sample size is 2. There is no explicit power analysis used. It is generally thought that an effect obtained in one monkey, even if replicated in hundreds of neurons should also be replicated in at least one additional monkey. Thus the standard size for an "experimental group" might be said to be two. However, occasions arise in which an ad hoc decision to use more animals is clearly appropriate, for example if results are not consistent. The concent of "experimental" groups and "control" groups is meaningless. In the study of each animal, one behavioral condition is typically the "experiment" and another condition the "control". The general guiding principle is always to use the minimal possible number of animals while validating all results through replication. For each monkey, we image nearly 1000 neurons, which is the sample size of neurons in each monkey in our study. The information of sample-size can be found in Subject section in Methods.

**Replicates**

- You should report how often each experiment was performed
- You should include a definition of biological versus technical replication
- The data obtained should be provided and sufficient information should be provided to indicate the number of independent biological and/or technical replicates
- If you encountered any outliers, you should describe how these were handled
- Criteria for exclusion/inclusion of data should be clearly stated
- High-throughput sequence data should be uploaded before submission, with a private link for reviewers provided (these are available from both GEO and ArrayExpress)

Please outline where this information can be found within the submission (e.g., sections or figure legends), or explain why this information doesn’t apply to your submission:

In this study, one experiment was performed per monkey, each with 2,250 stimulus conditions, each stimulus condition was repeated three times. The information of replicates can be found in the first and second paragraphs in Results and discussion and in the Visual stimuli section in Methods.

**Statistical reporting**

- Statistical analysis methods should be described and justified
- Raw data should be presented in figures whenever informative to do so (typically when N per group is less than 10)
- For each experiment, you should identify the statistical tests used, exact values of N, definitions of center, methods of multiple test correction, and dispersion and precision measures (e.g., mean, median, SD, SEM, confidence intervals; and, for the major substantive results, a measure of effect size (e.g., Pearson's r, Cohen's d)
- Report exact p-values wherever possible alongside the summary statistics and 95% confidence intervals. These should be reported for all key questions and not only when the p-value is less than 0.05.

Please outline where this information can be found within the submission (e.g., sections or figure legends), or explain why this information doesn’t apply to your submission:

ROC analysis was performed to evaluate the statistical significance, repeatability and reliability of the observed effects. This is documented in "Stability and reliability of the neuronal measurements" in Method section. Statistical tests were also performed to identify cells from the imaging data, documented in "Image data analysis" in Method section. The statistical significance of the stimulus selectivity is also documented (Fig 1d and 1f).

(For large datasets, or papers with a very large number of statistical tests, you may upload a single table file with tests, Ns, etc., with reference to sections in the manuscript.)

**Group allocation**

- Indicate how samples were allocated into experimental groups (in the case of clinical studies, please specify allocation to treatment method); if randomization was used, please also state if restricted randomization was applied
- Indicate if masking was used during group allocation, data collection and/or data analysis

Please outline where this information can be found within the submission (e.g., sections or figure legends), or explain why this information doesn’t apply to your submission:

The concept of "experimental group" is not meaningful as applied to nonhuman primate neuronal recording studies because typically two monkeys were used, and hence group size might be said to be 2. In our study, to determine the preference of a neuron to a stimulus, one might consider a stimulus as an "experiment", while the other stimuli are controls. The general guiding principle is to validate the results through replications.

**Additional data files (“source data”)**

- We encourage you to upload relevant additional data files, such as numerical data that are represented as a graph in a figure, or as a summary table
- Where provided, these should be in the most useful format, and they can be uploaded as “Source data” files linked to a main figure or table
- Include model definition files including the full list of parameters used
- Include code used for data analysis (e.g., R, MatLab)
- Avoid stating that data files are “available upon request”

Please indicate the figures or tables for which source data files have been provided:

All numerical data for the graphs and codes (decoding and ROC analysis codes) will be available in Github (link: https://github.com/leelabcnbc/sparse-coding-elife2018), as stated in the Data and Source codes section.
